# QuickStats

**Published:** 2014-01-17

**Authors:** FROM THE NATIONAL CENTER FOR HEALTH STATISTICS

**Figure:**
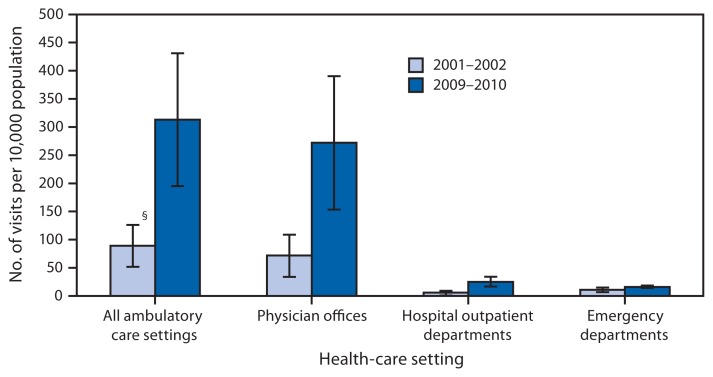
Rate* of Ambulatory Care Visits for Chronic Kidney Disease,^†^ by Health-Care Setting — United States, 2001–2002 and 2009–2010 * Per 10,000 population, based on 2-year annual averages. Rates were calculated using U.S. Census Bureau 2000-based postcensal civilian noninstitutionalized population estimates. ^†^ Defined as any listed diagnosis codes 403.00–403.91, 404.00–404.93, or 585 based on the *International Classification Of Diseases, Ninth Edition, Clinical Modification*. ^§^ 95% confidence interval.

From 2001–2002 to 2009–2010, the rate of ambulatory care visits overall for chronic kidney disease more than tripled in the United States, from 89 to 313 visits per 10,000 population. Visit rates increased for physician offices, from 72 to 272 per 10,000 population, and for hospital outpatient departments, from 6 to 25 per 10,000 population, but the chronic kidney disease visit rate for emergency departments did not change.

**Sources:** National Ambulatory Medical Care Survey, National Hospital Ambulatory Medical Care Survey. Available at http://www.cdc.gov/nchs/ahcd.htm.

**Reported by:** Anjali Talwalkar, MD, atalwalkar@cdc.gov; Kathleen Palso, MA.

